# β-Mannanase-catalyzed synthesis of alkyl mannooligosides

**DOI:** 10.1007/s00253-018-8997-2

**Published:** 2018-04-22

**Authors:** Johan Morrill, Anna Månberger, Anna Rosengren, Polina Naidjonoka, Pernille von Freiesleben, Kristian B. R. M. Krogh, Karl-Erik Bergquist, Tommy Nylander, Eva Nordberg Karlsson, Patrick Adlercreutz, Henrik Stålbrand

**Affiliations:** 1 Department of Biochemistry and Structural Biology, Lund University, PO Box 124, S-221 00 Lund, Sweden; 2 Department of Biotechnology, Lund University, PO Box 124, S-221 00 Lund, Sweden; 3 Department of Physical Chemistry, Lund University, PO Box 124, S-221 00 Lund, Sweden; 4 Centre for Analysis and Synthesis, Department of Chemistry, Lund University, PO Box 124, S-221 00 Lund, Sweden; 5 Novozymes A/S, Krogshøjvej 36, 2880 Bagsværd, Denmark

**Keywords:** β-Mannanase, Transglycosylation, Alcoholysis, Alkyl glycoside, Surfactant

## Abstract

β-Mannanases catalyze the conversion and modification of β-mannans and may, in addition to hydrolysis, also be capable of transglycosylation which can result in enzymatic synthesis of novel glycoconjugates. Using alcohols as glycosyl acceptors (alcoholysis), β-mannanases can potentially be used to synthesize alkyl glycosides, biodegradable surfactants, from renewable β-mannans. In this paper, we investigate the synthesis of alkyl mannooligosides using glycoside hydrolase family 5 β-mannanases from the fungi *Trichoderma reesei* (*Tr*Man5A and *Tr*Man5A-R171K) and *Aspergillus nidulans* (*An*Man5C). To evaluate β-mannanase alcoholysis capacity, a novel mass spectrometry-based method was developed that allows for relative comparison of the formation of alcoholysis products using different enzymes or reaction conditions. Differences in alcoholysis capacity and potential secondary hydrolysis of alkyl mannooligosides were observed when comparing alcoholysis catalyzed by the three β-mannanases using methanol or 1-hexanol as acceptor. Among the three β-mannanases studied, *Tr*Man5A was the most efficient in producing hexyl mannooligosides with 1-hexanol as acceptor. Hexyl mannooligosides were synthesized using *Tr*Man5A and purified using high-performance liquid chromatography. The data suggests a high selectivity of *Tr*Man5A for 1-hexanol as acceptor over water. The synthesized hexyl mannooligosides were structurally characterized using nuclear magnetic resonance, with results in agreement with their predicted β-conformation. The surfactant properties of the synthesized hexyl mannooligosides were evaluated using tensiometry, showing that they have similar micelle-forming properties as commercially available hexyl glucosides. The present paper demonstrates the possibility of using β-mannanases for alkyl glycoside synthesis and increases the potential utilization of renewable β-mannans.

**Electronic supplementary material:**

The online version of this article (10.1007/s00253-018-8997-2) contains supplementary material, which is available to authorized users.

## Introduction

Plant biomass has the potential to substitute fossil resources in numerous sectors. In this development, considerable interest is devoted to biotechnology for the sustainable production of biofuels and biochemicals (Cherubini 2010). Alkyl glycosides are non-toxic and biodegradable non-ionic surfactants suitable for many applications including detergents, cleaners, and personal care products (von Rybinski and Hill 1998). They consist of a hydrophobic alkyl chain linked by a glycosidic bond to a hydrophilic glycoside.

Glycoside hydrolases (GHs) can be used to synthesize alkyl glycosides from carbohydrates and alcohols under relatively mild reaction conditions (Ochs et al. 2011; van Rantwijk et al. 1999). Enzymatic synthesis of alkyl glycosides has several advantages over chemical synthesis in that it enables production of anomerically pure molecules, without the use of several protection and deprotection steps for chemoselectivity (van Rantwijk et al. 1999; von Rybinski and Hill 1998; Wang and Huang 2009). GH-catalyzed synthesis can be done either via thermodynamically controlled reversed hydrolysis or kinetically controlled transglycosylation (van Rantwijk et al. 1999).

**Fig. 1:**
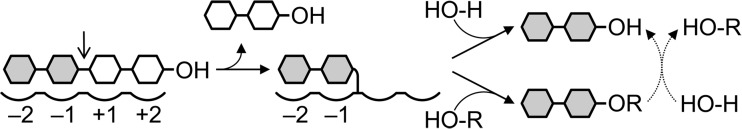
Simplified scheme illustrating the retaining double-displacement mechanism used by the β-mannanases in this study. To the left, mannotetraose (M_4_) is shown bound in subsites − 2 to + 2 with the reducing end bound in subsite + 2. β-Mannanases may bind M_4_ in multiple binding modes, e.g., from subsites − 3 to + 1 (Rosengren et al. 2012), resulting in other products. The enzyme performs a nucleophilic attack instead the glycosidic bond between subsites − 1 and + 1, indicated by the arrow, and releases the mannobiose unit in subsites + 1 and + 2 (white) while the mannobiose unit in subsites − 1 and − 2 (gray) forms a covalent intermediate with the enzyme. The covalent intermediate (in the center) can then be attacked by water (HO–H), leading to hydrolysis, or an alcohol (HO–R), leading to alcoholysis and the production of a glycosyl conjugate (alkyl mannobioside) shown to the right. The dashed arrows show possible secondary hydrolysis of the glycosyl conjugate

In the Carbohydrate-Active Enzymes (CAZy) database (http://www.cazy.org) (Lombard et al. 2014), GHs are classified into families based on sequence similarity, and some GH families have further been divided into subfamilies, e.g., GH family 5 (GH5) (Aspeborg et al. 2012). Families are classified into clans, where clan A is the largest (Davies and Sinnott 2008; Henrissat and Bairoch 1996). The active sites of endo-acting GHs, e.g., GH5 β-mannanases, contain several subsites where the monosaccharide moieties of the substrate bind. The subsites are labeled from −*n* to +*n*, where *n* is a positive integer, with −*n* situated towards the non-reducing end and +*n* towards the reducing end of the substrate (Fig. 1). Glycosidic bond cleavage occurs between monosaccharide moieties bound at the adjacent − 1 and + 1 subsites (Davies et al. 1997).

The GHs applicable for transglycosylation utilize a two-step catalytic mechanism which retains the configuration of the anomeric carbon (Sinnott 1990). The GH5 subfamily 7 (GH5_7) β-mannanases belonging to clan A studied in the present paper utilize this mechanism (Gilbert et al. 2008). The first step of the retaining mechanism is a nucleophilic attack on the anomeric carbon, which releases the leaving group and forms a covalent glycosyl-enzyme intermediate (Davies and Henrissat 1995; Zechel and Withers 2000). In the second step, a glycosyl acceptor, water in hydrolysis and another nucleophile in transglycosylation, performs a nucleophilic attack on the covalent intermediate which breaks the covalent bond, forming a new product (Fig. 1). Several GH5 β-mannanases have been shown to use saccharides as transglycosylation acceptors (Arcand et al. 1993; Coulombel et al. 1981; Couturier et al. 2013; Dias et al. 2004; Dilokpimol et al. 2011; Hakamada et al. 2014; Harjunpää et al. 1999; Hrmova et al. 2006; Larsson et al. 2006; Mizutani et al. 2012; Morrill et al. 2015; Puchart et al. 2004; Rosengren et al. 2012; Rosengren et al. 2014; Schröder et al. 2006; Wang et al. 2014). Alcohols acting as acceptors in combination with glycosyl donor substrates can generate alkyl glycosides (Adlercreutz 2017; Rosengren et al. 2014). In the presence of both water and alcohol, these acceptors compete to attack the covalent intermediate. Secondary hydrolysis of products may also occur (Fig. 1), and the synthesis and breakdown of several products are possible before equilibrium is reached (Sinnott 1990; van Rantwijk et al. 1999).

To date, alkyl glycoside synthesis with GHs has been carried out with, e.g., β-glucosidases (Lundemo et al. 2013; Papanikolaou 2001; Turner et al. 2007), β-mannosidases (Itoh and Kamiyama 1995), α-amylases (Damián-Almazo et al. 2008; Larsson et al. 2005; Moreno et al. 2010), xylanases (Jiang et al. 2004; Matsumura et al. 1996; Matsumura et al. 1997; Matsumura et al. 1999; Ochs et al. 2011), and β-glucanases (Akiba et al. 1999). However, little is known about alkyl glycoside synthesis catalyzed by endo-acting GHs attacking β-mannans which are among the most abundant polysaccharides in nature, e.g., constituting the major part of softwood hemicellulose (Ebringerová 2006; Lundqvist et al. 2003; Scheller and Ulvskov 2010). We have previously observed transglycosylation with β-mannanases using methanol and 1-butanol as acceptors (Rosengren et al. 2012; Rosengren et al. 2014). β-Mannanases have several potential and existing applications to increase the use of this interesting natural resource (Moreira and Filho 2008; Yamabhai et al. 2016). In the present paper, we address a novel approach—the application of β-mannanases in synthesis of alkyl glycosides using renewable β-mannan as donor substrate. Successful use of β-mannanases for enzymatic synthesis is especially interesting since the β-mannosidic bond is arguably the most difficult glycosidic bond to synthesize by chemical means (Gridley and Osborn 2000). Frequently, activated (e.g., nitrophenyl) sugars are used as donor substrates in transglycosylation reactions with *exo*-acting GHs as catalysts (Teze et al. 2015; Teze et al. 2014). Our approach in the current paper, however, is different. We are studying the use of a natural, renewable (non-activated) donor substrate (β-mannan) and endo-glycosidases (β-mannanases) as catalysts, allowing the synthesis of alkyl glycosides with longer sugar heads. Furthermore, in the present study, we expand the length of the acceptor alcohol used compared to previous studies (Rosengren et al. 2014) of β-mannanases.

With the aim to reveal the applicability of β-mannanases in alkyl mannooligoside synthesis, three fungal GH5 β-mannanases were selected based on previously observed alcoholysis capabilities with methanol and butanol as well as different product profiles due to different modes of mannotetraose (M_4_) attack: from *Trichoderma reesei*, *Tr*Man5A (Sabini et al. 2000) and its engineered subsite + 2 variant *Tr*Man5A-R171K (Rosengren et al. 2012), and from *Aspergillus nidulans*, *An*Man5C (Dilokpimol et al. 2011; Rosengren et al. 2014).

In this paper, we further study these three β-mannanases for the synthesis of alkyl mannooligosides using a longer-chain alcohol, i.e., hexanol, as acceptor (resulting in hexyl mannooligosides). A further novelty is the use of a natural β-mannan donor substrate. Mass spectrometry was used as a novel screening method to estimate the initial degree of alcoholysis products (DA) early in reactions where a significant amount of donor substrate remains. DA reflects the fraction of total products that are alkyl mannooligosides. One β-mannanase (*Tr*Man5A) was selected for further optimization on the basis of highest DA values and stable product during prolonged incubations. Using pre-hydrolyzed β-mannan as donor substrate, a sufficiently large reaction was set up to allow product characterization. The synthesized hexyl mannooligosides were separated and quantified using reversed-phase liquid chromatography, their structures were characterized, and their basic surfactant properties were determined.

## Materials and methods

### Cloning, expression, and purification

*Tr*Man5A was produced in the host *T. reesei* as described earlier (Hägglund et al. 2003) and was lyophilized. An aliquot of the obtained powder was solubilized in 50 mM sodium acetate buffer (pH 5.3). To remove saccharides present in the enzyme powder, the solution was concentrated by centrifugation in a spin column with 10 kDa cutoff followed by dilution in the buffer and this procedure was repeated several times.

For production of *Tr*Man5A-R171K, *Pichia pastoris* X-33 cells transformed with the constructed plasmid encoding *Tr*Man5A-R171K as described previously (Rosengren et al. 2012) were cultured and expressed as described in the EasySelect™ *Pichia* expression kit manual (Invitrogen, Lidingö, Sweden). The cells were streaked on an agar plate containing yeast extract peptone dextrose (YPD) medium with 100 μg/mL Zeocin™ and incubated at 30 °C for 3 days. One colony was transferred to a 250-mL baffled Erlenmeyer flask containing 50 mL buffered glycerol-complex medium (BMGY) and incubated at 30 °C and 250 rpm to an OD_600_ of 2.0. The culture was then centrifuged for 30 min, the supernatant was discarded, and the pellet was dissolved in 400 mL buffered methanol-complex medium (BMMY) to an OD_600_ of 1.0 in two 2-L baffled Erlenmeyer flasks for expression. The expression culture was incubated at 30 °C and 250 rpm with methanol added every 24 h to a final concentration of 0.5%. Culture enzyme activity was monitored with the β-mannanase activity assay described below. After 6 days of expression, the supernatants were harvested by centrifugation and the cell pellets were discarded. The supernatant was concentrated and changed to 10 mM Tris-HCl buffer (pH 7.8) using spin columns with 10 kDa cutoff. Anion exchange chromatography was performed on a BioLogic DuoFlow FPLC^®^ System (Bio-Rad, Hercules, CA, USA) with a 6-mL Resource Q anion exchange column (Amersham Pharmacia Biotech, Uppsala, Sweden). The flow rate was 1 mL/min, and 40 fractions of 5 mL each were collected over a sodium chloride gradient of 0–0.5 M. The purest fractions, evaluated with sodium dodecyl sulfate-polyacrylamide gel electrophoresis (SDS-PAGE) (Rosengren et al. 2012), were pooled and changed to 50 mM sodium acetate buffer (pH 5.3) using a spin column with 10 kDa cutoff.

For production of *An*Man5C, *P. pastoris* X-33 cells containing the gene encoding the enzyme were obtained from the Fungal Genetics Stock Center (FGSC), School of Biological Science, University of Missouri (Kansas City, MO, USA), with accession number 10106 (AN6427.2). The gene was previously cloned from complementary DNA of *A. nidulans* by others (Bauer et al. 2006). The cells were cultured and expressed, and the supernatant was harvested in the same way as described for *Tr*Man5A-R171K above. *An*Man5C was purified as described previously (Rosengren et al. 2014). The supernatant was concentrated by centrifugation in a spin column with 10 kDa cutoff. His-tag purification was performed with a 1-mL Ni-NTA Superflow cartridge according to the manufacturer’s recommendations (Qiagen, Hilden, Germany). Fractions with pure protein, verified by SDS-PAGE, were pooled and changed to 50 mM sodium acetate buffer (pH 5.5), by centrifugation using a 10-kDa cutoff spin column.

Protein concentrations were determined using a NanoDrop^®^ ND-1000 spectrophotometer (Saveen Werner, Malmö, Sweden) by measuring absorbance at 280 nm as described previously for *Tr*Man5A and *Tr*Man5A-R171K (Rosengren et al. 2012) as well as for *An*Man5C (Rosengren et al. 2014).

As assay buffers, 50 mM sodium acetate buffer (pH 5.3) for *Tr*Man5A and *Tr*Man5A-R171K and 50 mM sodium acetate buffer (pH 5.5) for *An*Man5C were used if not otherwise stated. The 1-hexanol was dried with a 3 Å molecular sieve (Sigma-Aldrich, St. Louis, MO, USA) for at least 24 h before use.

### β-Mannanase activity assay

β-Mannanase activity was assayed with 0.5% (*w*/*v*) locust bean galactomannan (LBG) (Sigma-Aldrich) in buffer using a scaled-down version of the 3,5-dinitrosalicylic acid (DNS) method described previously (Stålbrand et al. 1993). Six microliters of adequately diluted enzyme was mixed with 54 μl of 0.5% (*w*/*v*) LBG in a 96-well plate and incubated for 10 min at 37 °C in a PTC-200 thermocycler (Bio-Rad). One hundred twenty microliters DNS was added to the mixture to stop the reaction. The mixture was heated at 95 °C for 5 min and subsequently cooled in the thermocycler before the absorbance was measured at 540 nm in a SpectraCount™ plate reader (Packard, Meriden, CT, USA). The concentration of reducing ends was calculated from a standard curve of mannose.

### β-Mannanase stability in alcohol

The stability of the three β-mannanases was tested in 25, 50, and 75% (*v*/*v*) methanol; in 5 and 25% (*v*/*v*) 1-hexanol; and without any alcohol. The three enzymes were each diluted with assay buffer and alcohol at the abovementioned concentrations prior to incubation. The stability test was performed at room temperature and at 37 °C. After 0, 2, 6, and 24 h of incubation, samples were taken and residual β-mannanase activity was assayed in each sample as described above.

### Alcoholysis with M_4_ and methanol

Alcoholysis with methanol was performed by incubating 2 μM of each enzyme with 5 mM mannotetraose (M_4_) and 25% (*v*/*v*) methanol in 20 mM buffer at 37 °C for 4 h (Rosengren et al. 2012). Samples were taken every hour and analyzed with matrix-assisted laser desorption/ionization time-of-flight mass spectrometry (MALDI-TOF MS) as described previously (Hekmat et al. 2010; Rosengren et al. 2012). 0.5 μL of each reaction mixture was applied and quickly dried by heating on a stainless steel target plate. Then, 0.5 μL matrix (10 mg/mL 2,5-dihydroxybenzoic acid (DHB) in water) was applied over the sample and quickly dried by heating. A 4700 Proteomics Analyzer (Applied Biosystems, Foster City, CA, USA) in positive reflector mode was used with a laser intensity of 5000. Fifty subspectra with 20 shots on each were accumulated from a sample to generate a spectrum. Data Explorer version 4.5 (Applied Biosystems) was used for data analysis.

### Alcoholysis with M_4_ and 1-hexanol

Alcoholysis with 1-hexanol was performed in the same way as described with methanol, except that 25% (*v*/*v*) 1-hexanol was used instead of methanol, and prior to the sampling of the reaction mixture, the tube was shaken to mix the two phases. Reactions with *Tr*Man5A were also performed with varying enzyme concentrations (0.2, 2, and 4 μM) and M_4_ concentrations (5, 25, and 50 mM). Duplicate incubations were analyzed with MALDI-TOF MS as described above, except that the samples were diluted 10-fold in Milli-Q water before being applied on the target plate due to the presence of hexanol. In addition, reactions with 5, 25, and 50 mM M_4_ were analyzed with high-performance anion exchange chromatography with pulsed amperometric detection (HPAEC-PAD) in order to analyze the rate of M_4_ degradation in these reactions, using an ICS-5000 system and a CarboPac PA200 column (Thermo Fisher Scientific, Waltham, MA, USA).

### Calculation of degree of alcoholysis products

In alcoholysis reactions with both alcohol and water present together with a donor saccharide, both hydrolysis products (oligosaccharides) and alkyl glycosides are likely formed. The product formation (here analyzed with MALDI-TOF MS) can be described by the DA, reflecting the fraction of total products that are alkyl mannooligosides. To make an initial estimation of the DA for a given reaction, the peak areas of alkyl mannooligosides and mannooligosaccharides that have accumulated from the start of a reaction to the sampling time were obtained from the same MALDI-TOF MS spectrum. Since different compounds are expected to have different response factors in MALDI-TOF MS (Duncan et al. 2008), the DA determined in this way does not reflect the absolute concentration relation of products. However, DA analysis can still serve as a straightforward initial method to compare different enzymes and/or reaction conditions. In this case, to estimate DA in reactions with M_4_ as donor substrate, the MALDI-TOF MS peak areas of the alcoholysis products (alkyl mannooligosides) and oligosaccharide products were determined. Hexyl mannoside and mannose (M_1_) were excluded in the present study due to being minor reaction products (very low MALDI-TOF MS response), and M_4_ was excluded due to being the reaction donor substrate. DA was then calculated from MALDI-TOF MS peak areas according to Eq. (1).

1 $$ \mathrm{DA}=\frac{\mathrm{Total}\ \mathrm{area}\ \mathrm{of}\ \mathrm{alkyl}\ \mathrm{mannooligosides}}{\mathrm{Total}\ \mathrm{area}\ \mathrm{of}\ \mathrm{oligosaccharide}\ \mathrm{products}\ \mathrm{and}\ \mathrm{alkyl}\ \mathrm{mannooligosides}} $$

The initial DA values after 1 h of incubation were calculated for reactions with 2 μM *Tr*Man5A, *Tr*Man5A-R171K, or *An*Man5C with 5 mM M_4_ and 25% (*v*/*v*) methanol or 1-hexanol, as well as during the course of further reactions with 1-hexanol.

### Separation and quantification of hexyl mannooligosides

To separate and quantify the synthesized hexyl mannooligosides, the reaction was performed as described above with 25% (*v*/*v*) 1-hexanol, 0.2 μM *Tr*Man5A, and 25 mM M_4_ for up to 8 h. Samples were taken every 2 h, and the reaction was stopped by heating the samples at 95 °C for 5 min. The samples were then diluted fourfold with water and acetonitrile (ACN) to 20% (*v*/*v*) ACN. The diluted samples were analyzed on an UltiMate 3000 high-performance liquid chromatography (HPLC) system (Thermo Fisher Scientific) using an Acclaim RSLC 120 C_18_ column with a Corona charged aerosol detector (CAD). Five microliters of each sample was injected and separated with a 0.5 mL/min mobile phase composed of 85% of 0.1% (*v*/*v*) acetic acid in water and 15% ACN over 20 min at a column temperature of 40 °C. Concentrations of hexyl mannooligosides were estimated using a standard curve of hexyl-β-d-maltoside (Sigma-Aldrich). Fractions were collected at chromatogram peaks during HPLC separation and analyzed with MALDI-TOF MS in order to determine peak identities.

### Calculation of alcoholysis/hydrolysis ratio

In order to more accurately and mechanistically describe the competition between alcohol and water in the reaction mixture with *Tr*Man5A, the alcoholysis/hydrolysis ratio (*r*_A_/*r*_H_) was calculated as described in the literature (van Rantwijk et al. 1999). In kinetically controlled transglycosylation reactions with alcohol as the main (non-water) acceptor, *r*_A_/*r*_H_ describes the prevalence of the covalent intermediate being attacked by alcohol as opposed to water. Due to hexyl mannobioside (hexyl-M_2_) being the dominant alkyl mannooligoside produced by *Tr*Man5A, *r*_A_/*r*_H_ was calculated according to Eq. (2) based on HPLC and HPAEC-PAD quantifications of hexyl-M_2_ and mannobiose (M_2_), respectively. Hydrolysis of M_4_ generates two M_2_ molecules, while alcoholysis generates M_2_ and hexyl-M_2_ in equimolar amounts (Fig. 1). Thus, the denominator is calculated by subtracting [alkyl-M_2_] from [M_2_] and dividing the obtained value by 2.

2 $$ \frac{r_{\mathrm{A}}}{r_{\mathrm{H}}}=\frac{\left[\mathrm{alkyl}-{\mathrm{M}}_2\right]}{\left(\left[{\mathrm{M}}_2\right]-\left[\mathrm{alkyl}-{\mathrm{M}}_2\right]\right)/2} $$

From the initial *r*_A_/*r*_H_, the theoretical yield (*η*) was extrapolated according to Eq. (3) (van Rantwijk et al. 1999) and compared with experimental yield to assess secondary hydrolysis of alcoholysis products.

3 $$ \eta =\frac{r_{\mathrm{A}}/{r}_{\mathrm{H}}}{1+{r}_{\mathrm{A}}/{r}_{\mathrm{H}}} $$

The selectivity factor (*S*_c_) indicating the enzyme’s selectivity for the alcohol (Adlercreutz 2017; Hansson and Adlercreutz 2001) was also calculated from the initial *r*_A_/*r*_H_ according to Eq. (4). In the case of 1-hexanol, the concentrations of 1-hexanol (5.9 g/L, equal to 58 mM) and water (55 M) in the water phase of the reaction mixture were used to calculate *S*_c_.

4 $$ {S}_{\mathrm{c}}=\frac{r_{\mathrm{A}}}{r_{\mathrm{H}}}\times \frac{\left[\mathrm{water}\right]}{\left[\mathrm{alcohol}\right]} $$

### Preparative synthesis and purification of hexyl mannooligosides

To prepare sufficient amounts of hexyl mannooligosides for characterization, the alcoholysis reaction was scaled up. Mannooligosaccharides were prepared by hydrolysis of 4 g ivory nut mannan (INM) (Megazyme, Bray, Ireland) for 4 h by 0.25 μM of the GH26 β-mannanase from *Podospora anserina*, *Pa*Man26A (Couturier et al. 2013; von Freiesleben et al. 2016), in 400 mL of 20 mM ammonium acetate buffer (pH 5.3) at 40 °C in a 2-L baffled Erlenmeyer flask with shaking at 150 rpm. The oligosaccharide composition of the resulting hydrolysate was determined with HPAEC-PAD, and the hydrolysate was lyophilized. Hydrolysate with 25 mM of M_4_ was then used as donor substrate in a 35-mL reaction with 25% (*v*/*v*) 1-hexanol and 0.2 μM *Tr*Man5A in 20 mM sodium acetate buffer (pH 5.3). The reaction was performed for 8 h at 37 °C and then stopped by boiling for 5 min. Hexyl mannooligosides in the sample were quantified with HPLC as described above. The obtained hexyl mannooligosides were then purified with preparative HPLC using a Waters Symmetry C_18_ Prep column using a 1260 Infinity system (Agilent Technologies, Santa Clara, CA, USA), with a 10–45% gradient of ACN versus 0.1% formic acid in water over 9 min at room temperature, followed by a steeper gradient of 45–90% ACN over 2 min and, finally, a wash step with 90% ACN for 2 min. Fractions were collected during the entire separation and analyzed with MALDI-TOF MS to identify fractions containing hexyl mannooligosides. The identified fractions were then pooled, lyophilized, and redissolved in 25 μl Milli-Q water.

### Structural characterization of hexyl mannooligosides

For structural analysis, an aliquot of the lyophilized hexyl mannooligoside mixture synthesized above was analyzed with MALDI-TOF MS as described above. Peaks corresponding to hexyl mannooligosides were then fragmented with MALDI-TOF/TOF tandem MS. From each precursor mass, to generate a spectrum, 10 subspectra were collected in positive reflector mode with 125 shots per subspectrum at a laser intensity of 6000.

Further structural analysis was carried out with nuclear magnetic resonance (NMR) spectroscopy. Before analysis, the lyophilized sample of hexyl mannooligosides was dissolved in 500 μl of 99.8% D_2_O, equilibrated at room temperature overnight, lyophilized, and redissolved and equilibrated overnight again in 500 μl of 99.8% D_2_O. ^1^H, ^13^C, correlation spectroscopy (COSY), total correlation spectroscopy (TOCSY), heteronuclear multiple-bond correlation (HMBC), and heteronuclear single quantum coherence (HSQC) NMR spectra were recorded at 10 °C and a ^1^H spectrum also at 25 °C, on an Bruker Avance III spectrometer (Bruker, Billerica, MA, USA) at 500.17 and 125.78 MHz for ^1^H and ^13^C, respectively. Chemical shifts were given in ppm relative to tetramethylsilane (TMS) as external standard. Additionally, ^1^H NMR was used to quantify the hexyl-M_2_ and hexyl mannotrioside (hexyl-M_3_) in the sample by comparing it to reference samples of hexyl-β-d-maltoside. The same sample was also analyzed and quantified with HPLC, using hexyl-β-d-maltoside as standard.

### Determination of critical micelle concentration

In order to evaluate the surfactant properties of the synthesized hexyl mannooligosides, surface tension was measured as a function of hexyl mannooligoside concentration using a PAT-1 Drop and Bubble Shape Tensiometer (SINTERFACE Technologies, Berlin, Germany). This technique is based on analyzing the shape of the drop or bubble as described in the literature (Berry et al. 2015; Javadi et al. 2013). The shape is determined by the surface tension that strives to make a spherical drop and gravity forces that elongate the drop, which can be described by the Young-Laplace equation (Berry et al. 2015). The profile of the pendant drop was captured by a charge-coupled device (CCD) camera. To record data on small sample volumes, a 100-μl syringe with a needle that has an external diameter of 1.0 mm was fitted to the instrument and the sample was ejected manually. A typical drop volume was 5 μl. Since the technique is non-destructive, the same solution was reused for the following measurements, starting with the highest concentration and diluting to obtain the necessary concentration for the next point in the curve. If needed, the sample was freeze-dried between measurements. Measurements were performed at 22 °C for 350 s each and at least twice for every concentration. For comparison, surface tension curves were obtained in the same way with hexyl-β-d-glucoside and hexyl-β-d-maltoside individually and in a mixture with a 0.53:1 mole ratio.

## Results

### β-Mannanase stability in the presence of alcohol

To see if *Tr*Man5A, *Tr*Man5A-R171K, and *An*Man5C retained their β-mannanase activity in the presence of alcohols, enzyme stability was evaluated with several concentrations of methanol and 1-hexanol, with activity assayed at regular time intervals. The three enzymes retained 80–100% of initial activity over 24 h at 37 °C with 25% methanol or 5% 1-hexanol (Fig. S1). The enzymes were moderately stable with 25% 1-hexanol, retaining 75–90% of initial activity for at least 6 h. Higher methanol concentrations decreased stability further, with 75% methanol deactivating all three enzymes after 2 h.

### Alcoholysis with M_4_ and methanol

**Fig. 2:**
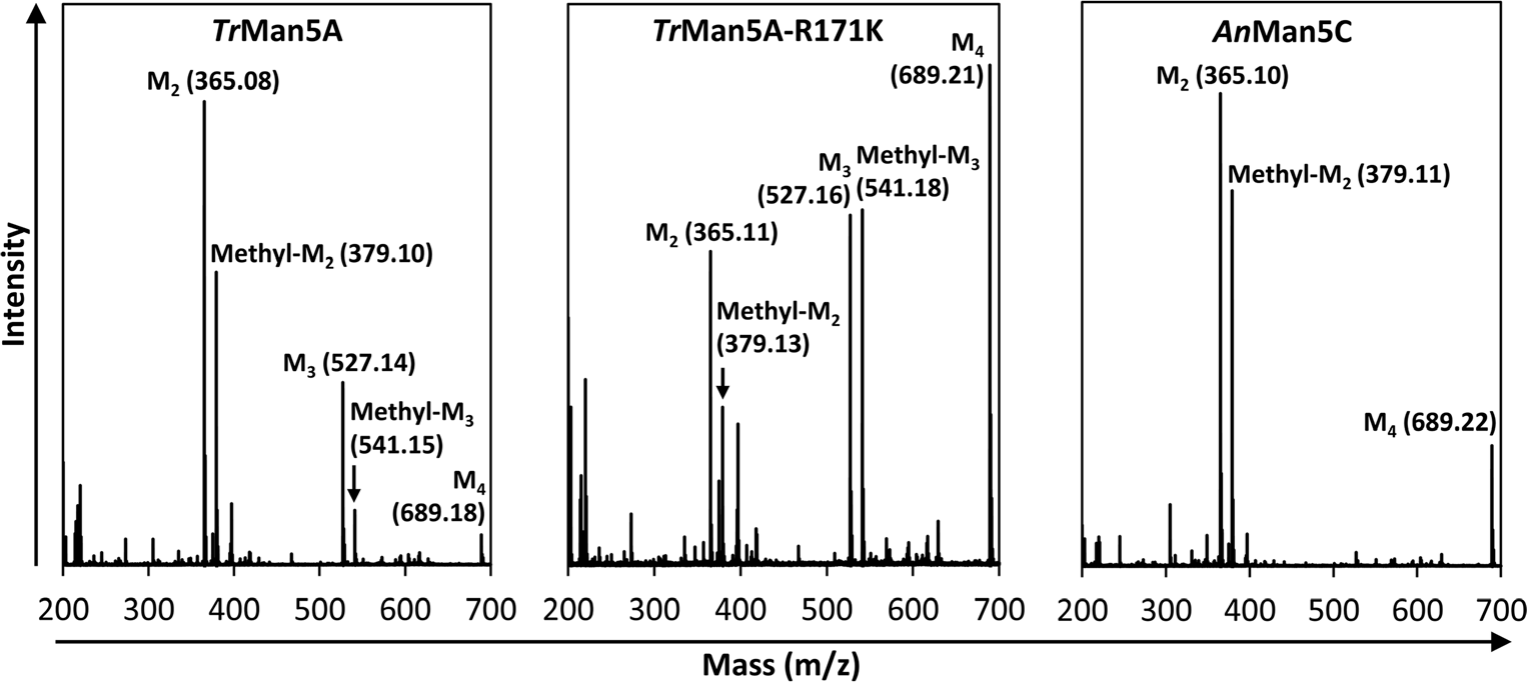
MALDI-TOF-MS spectra of alcoholysis reaction with methanol after 1 h. Peaks correspond to experimentally determined monoisotopic masses of sodium adducts of present mannooligosaccharides and methyl mannooligosides. The theoretical monoisotopic sodium adduct masses of these compounds are as follows: M_1_, 203.05; M_2_, 365.11; M_3_, 527.16; M_4_, 689.21; methyl-M_1_, 217.07; methyl-M_2_, 379.12; and methyl-M_3_, 541.17

In order to screen the enzymes’ capacity to catalyze alcoholysis, each enzyme was incubated with 5 mM M_4_ and 25% (*v*/*v*) methanol at 37 °C for up to 4 h. Alcoholysis products (methyl mannooligosides) and oligosaccharide products were detected with MALDI-TOF MS. After 1 h, the dominating product for both *Tr*Man5A and *An*Man5C was M_2_ followed by methyl mannobioside (methyl-M_2_) (Fig. 2), with *Tr*Man5A also producing some mannotriose (M_3_) and methyl mannotrioside (methyl-M_3_). The dominating products for *Tr*Man5A-R171K are M_3_ and methyl-M_3_ followed by M_2_ and methyl-M_2_. This is consistent with the observed subsite binding mode preferences with M_4_ for *Tr*Man5A and *Tr*Man5A-R171K (Rosengren et al. 2012). The R171K substitution in the + 2 subsite of *Tr*Man5A was previously shown to reduce the frequency of binding modes involving the + 2 subsite (Rosengren et al. 2012).

To advance the evaluation of alcoholysis with methanol by the three enzymes, the initial DA in the above reactions with methanol and M_4_ was calculated based on MALDI-TOF MS peak areas of alcoholysis products and oligosaccharide products according to Eq. (1) (Table 1). DA values reflect the fraction of total products that are alcoholysis products and allow comparison between enzymes as described in the “Materials and methods” section. The most effective enzymes to perform alcoholysis with M_4_ and methanol were *Tr*Man5A-R171K and *An*Man5C with initial DA values after 1 h of 0.43 and 0.42, respectively, followed by *Tr*Man5A with a DA of 0.33 (Table 1). In addition, minor amounts (< 2% of total product peak area) of saccharides with degree of polymerisatio (DP) 5–9 were detected in incubations with *Tr*Man5A and *An*Man5C, indicating transglycosylation with saccharides as acceptors, but not with *Tr*Man5A-R171K, in line with previous studies (Dilokpimol et al. 2011; Rosengren et al. 2012). The reactions were further followed during 4 h with sampling every hour, showing differences in DA during the course of the reactions. Both *Tr*Man5A and *An*Man5C show a maximum DA at 1 h. However, the DA beyond 1 h was almost constant for *Tr*Man5A and *Tr*Man5A-R171K while it dropped distinctly (> 10-fold) for *An*Man5C, possibly indicating secondary hydrolysis of the alcoholysis products.

**Table 1:** Degree of alcoholysis products (DA) (average of three MALDI-TOF MS spectra ± standard deviation) after 1 h of incubation with 5 mM M_4_, 25% (*v*/*v*) methanol or 1-hexanol, and 2 μM of *Tr*Man5A, *Tr*Man5A-R171K, or *An*Man5C

Enzyme	DA (methanol)	DA (1-hexanol)
*Tr*Man5A	0.33 ± 0.04	0.026 ± 0.003
*Tr*Man5A-R171K	0.43 ± 0.01	0.010 ± 0.001
*An*Man5C	0.42 ± 0.01	0.004 ± 0.001

DA values were calculated from MALDI-TOF MS peak areas according to Eq. (1)

### Alcoholysis with M_4_ and 1-hexanol

In order to screen and evaluate alcoholysis capacity with a longer-chain alcohol, 2 μM of each of the three enzymes was incubated with 5 mM M_4_ and 25% (*v*/*v*) 1-hexanol for up to 4 h. Alcoholysis products (hexyl mannooligosides) and oligosaccharides were detected with MALDI-TOF MS, and DA was calculated in the same way as with methanol above according to Eq. (1). The dominating alcoholysis products were of the same mannooligoside DP as those obtained from alcoholysis with methanol, with *Tr*Man5A producing mainly hexyl mannobioside (hexyl-M_2_) and some hexyl mannotrioside (hexyl-M_3_) (Fig. S2), *Tr*Man5A-R171K mainly hexyl-M_3_ and some hexyl-M_2_, and *An*Man5C exclusively hexyl-M_2_. Oligosaccharides with DP 5–9 were again detected in minor amounts (< 2% of total product MALDI-TOF MS peak area) with *Tr*Man5A and *An*Man5C but not with *Tr*Man5A-R171K. However, the DA values for all three enzymes were significantly lower than corresponding values for methyl mannooligosides produced by the same enzymes (Table 1). After 1 h of incubation, *Tr*Man5A had the highest DA followed by *Tr*Man5A-R171K and *An*Man5C (Table 1). Again *An*Man5C showed a drop in DA (> 10-fold) over 4 h of incubation, while *Tr*Man5A and *Tr*Man5A-R171K had almost stable DA values. On the basis of having the highest initial DA with 1-hexanol among the assayed enzymes, *Tr*Man5A was chosen for further studies of alcoholysis with 1-hexanol.

**Fig. 3:**
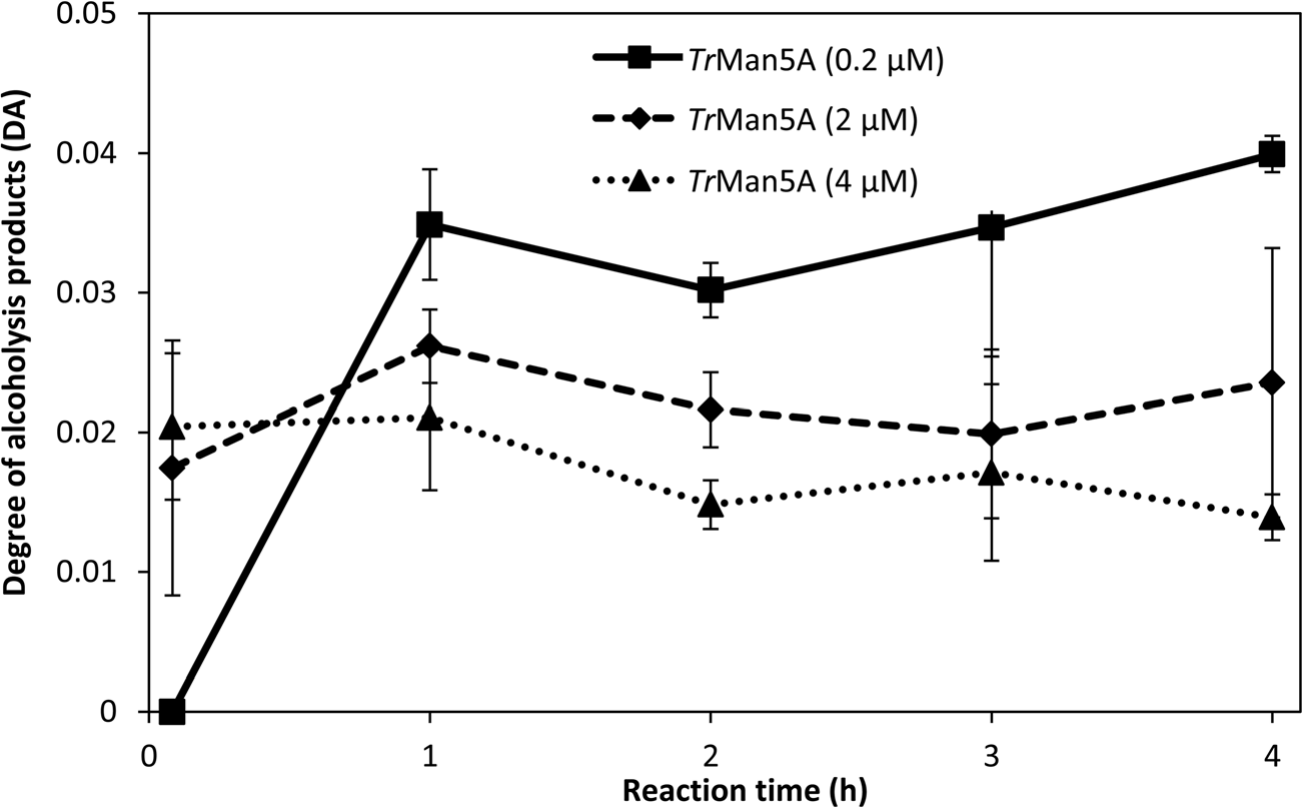
Degree of alcoholysis products (DA) over 0–4 h of incubations with 5 mM M_4_, 25% (*v*/*v*) 1-hexanol, and 0.2, 2, or 4 μM *Tr*Man5A. Error bars represent deviations between duplicate samples

Next, in order to optimize reaction conditions, the effect of varying *Tr*Man5A concentrations on DA was studied, using 5 mM M_4_ and 25% (*v*/*v*) 1-hexanol. Here, a lower enzyme concentration (0.2 μM) resulted in a higher DA compared to higher enzyme loads after 1 h (Fig. 3) where significant amounts of M_4_ remained. The DA increased up to 1 h with 0.2 μM *Tr*Man5A with a slight increase in DA over time during the course of the reaction, and the highest DA was observed after 4 h of incubation (Fig. 3). A slight decrease in DA was observed with the highest *Tr*Man5A concentration (4 μM) with increased incubation time, which could potentially be a result of secondary hydrolysis (Fig. 3). *Tr*Man5A at a concentration of 0.2 μM resulted in the highest DA after 1 h and a stable DA with increasing incubation time, and this *Tr*Man5A concentration was therefore used in subsequent reactions.

Further optimization of reaction conditions was performed by varying the concentration of the donor substrate, M_4_. HPAEC-PAD quantification of the apparent rate of M_4_ degradation was used in combination with DA values calculated from MALDI-TOF MS peak areas (Eq. (1)) in order to estimate hexyl mannooligoside product yields with 5, 25, or 50 mM M_4_. The use of 5 mM M_4_ resulted in substrate depletion after 1 h, whereas the reaction continued with the higher concentrations. After 4 h of incubation, the reactions with 5 and 25 mM M_4_ had similar DA values, but a higher rate of M_4_ conversion was observed with 25 mM M_4_ (Table 2). This suggests a higher hexyl mannooligoside yield with 25 mM M_4_. The DA was lower with 50 mM M_4_, possibly as a result of increased transglycosylation with saccharides as acceptors as indicated by higher MALDI-TOF MS detection of oligosaccharides with DP 5–9. After 4 h of incubation, saccharides with DP > 4 represented 1.4, 8.6, and 15% of total product peak area with 5, 25, and 50 mM M_4_, respectively. Also, with 25 and 50 mM M_4_, substantial amounts of M_4_ remained after 4 h, indicating the possibility of higher hexyl mannooligoside production if the reaction would be prolonged. Therefore, an extended reaction time of 8 h with 25 mM M_4_ was analyzed, and the reaction followed the same profile as the 4-h reaction, while the DA increased further as M_4_ was fully consumed (Table 2). Based on these results, an M_4_ concentration of 25 mM and a reaction time of up to 8 h were selected for reaction scale-up as well as HPLC separation and quantification of hexyl mannooligosides (see the next section).

**Table 2:** Rates of mannotetraose (M_4_) consumption and degree of alcoholysis products (DA) (average ± deviation between duplicate samples) at 1 and 4 h for alcoholysis reactions with 0.2 μM *Tr*Man5A, 25% (*v*/*v*) 1-hexanol, and 5, 25, or 50 mM M_4_. The lack of rate at 4 h with 5 mM M_4_ is due to the M_4_ being consumed after 1 h

[M_4_] (mM)	M_4_ rate at 1 h (μM/min)	M_4_ rate at 4 h (μM/min)	DA at 1 h	DA at 4 h	DA at 8 h
5	95 ± 4.7	–^a^	0.051 ± 0.016	0.043 ± 0.010	–^b^
25	123 ± 5.2	66 ± 10	0.022 ± 0.005	0.040 ± 0.002	0.059 ± 0.009
50	120 ± 84	60 ± 44	–^c^	0.021 ± 0.006	–^b^

Twenty-five millimolars of M_4_ was selected for prolonged (8-h) reaction (rightmost column)

^a^M_4_ consumed

^b^Reaction not carried out

^c^No hexyl mannosides detected

### Purification and quantification of hexyl mannooligosides

**Fig. 4:**
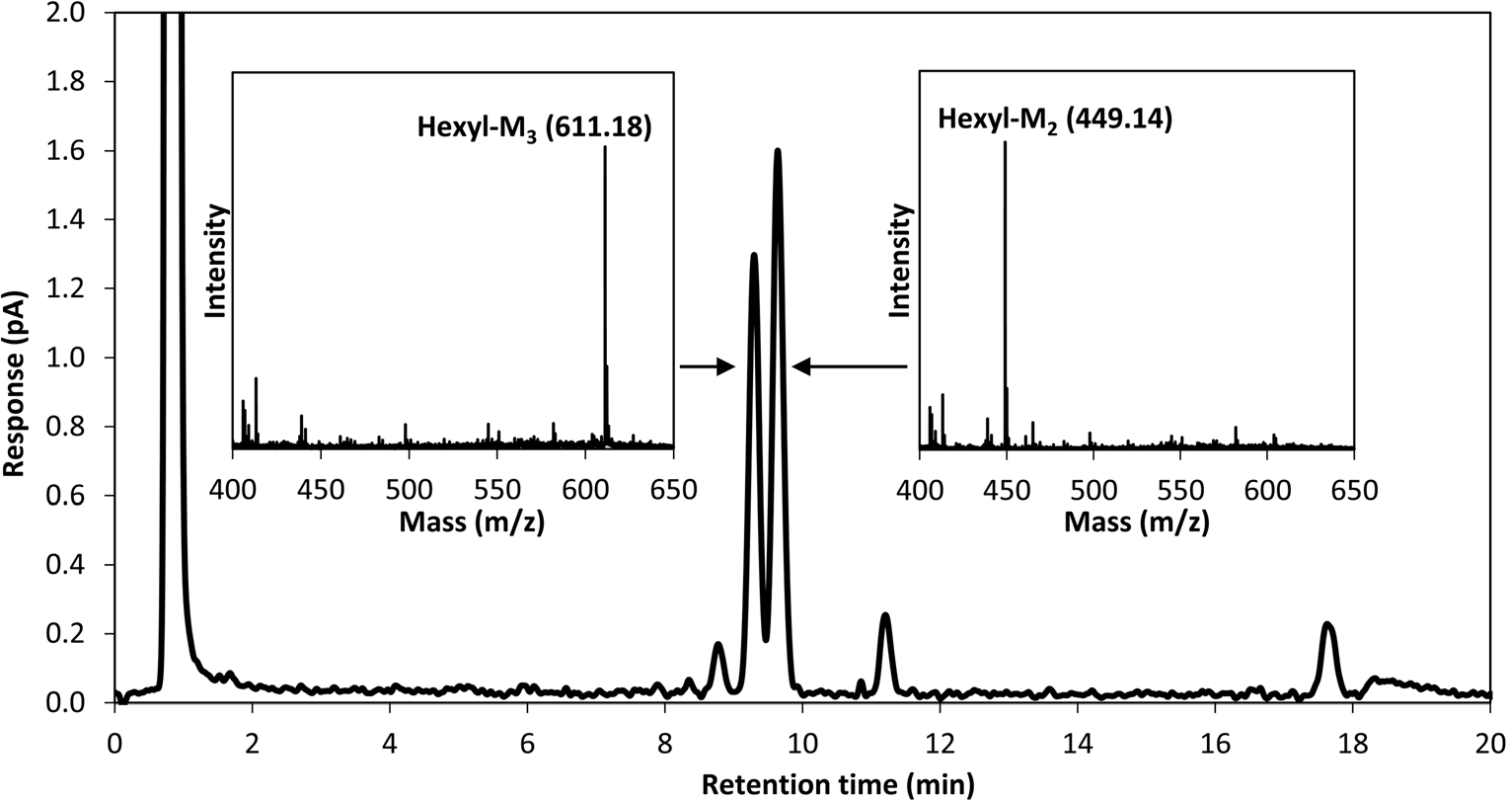
Chromatogram showing separation of hexyl mannooligosides with reversed-phase HPLC using a C_18_ column. The two inserts show MALDI-TOF MS spectra with peak mass identifications corresponding to monoisotopic sodium adduct masses of hexyl-M_3_ (theoretical mass 611.25) and hexyl-M_2_ (theoretical mass 449.20)

**Fig. 5:**
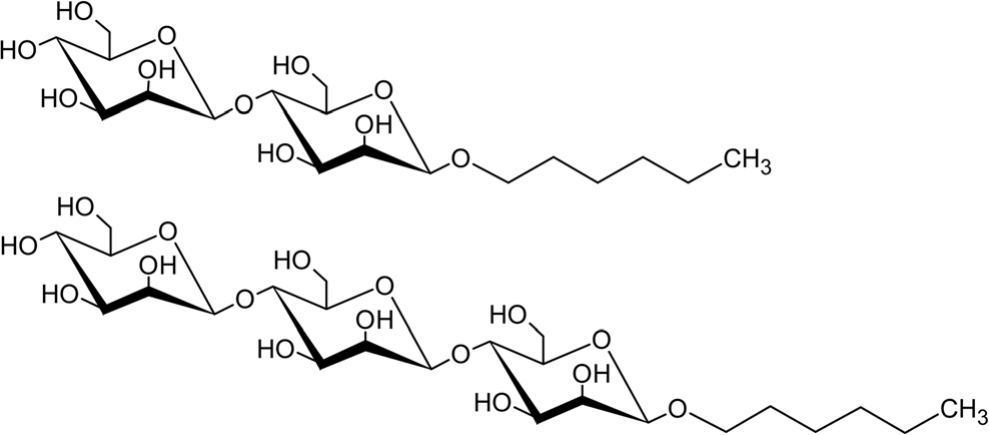
The predicted structure of hexyl β-d-mannobioside (top) and hexyl β-d-mannotrioside (bottom)

Reversed-phase (C_18_) HPLC with hexyl β-d-maltoside as standard was used to analyze reaction mixtures with 0.2 μM *Tr*Man5A, 25 mM M_4_, and 25% (*v*/*v*) 1-hexanol to more accurately separate and quantify hexyl mannooligosides produced by alcoholysis with *Tr*Man5A. Analytical separation of hexyl-M_2_ and hexyl-M_3_ was obtained with HPLC as confirmed with MALDI-TOF MS peak mass identification (*m*/*z* values of 449.14 and 611.18 for hexyl-M_2_ and hexyl-M_3_, respectively) (Fig. 4). Retaining β-mannanases are unequivocally expected to yield transglycosylation products (in this case, hexyl mannooligosides) with the same β-anomeric configuration as the donor substrate (Harjunpää et al. 1995; Sinnott 1990). Thus, the expected β-configured structures of the synthesized hexyl-M_2_ and hexyl-M_3_ are shown in Fig. 5. When the M_4_ was fully consumed after 8 h of incubation (DA value 0.059, Table 2), the concentration of the dominant alcoholysis product (hexyl-M_2_) was 0.48 mM, corresponding to a yield of 1.9% based on the initial M_4_ concentration.

Based on the M_2_ and hexyl-M_2_ concentrations after 2 h of incubation (8.1 and 0.13 mM, respectively), the *r*_A_/*r*_H_ was calculated according to Eq. (2), reflecting the competition between alcohol and water in attacking the covalent intermediate. Under these conditions (i.e., 0.2 μM *Tr*Man5A and 25 mM M_4_), the *r*_A_/*r*_H_ of *Tr*Man5A with 1-hexanol (25% (*v*/*v*)) was 0.033, corresponding to a theoretical alcoholysis product yield of 3.2% (Eq. (3)). Since the experimentally determined yield was 1.9%, the difference could possibly be the result of secondary hydrolysis (van Rantwijk et al. 1999). Although DA remained stable during prolonged incubations at lower M_4_ concentrations (Fig. 3), it is possible that secondary hydrolysis might become more prominent at higher M_4_ concentrations, assuming that hexyl mannooligosides would then be produced in higher amounts. With the assumption that the reaction occurs in the water phase (1-hexanol concentration 5.9 g/L or 58 mM), the enzyme’s *S*_c_ for 1-hexanol was calculated (Eq. (4)). *S*_c_ describes the relative preference of an enzyme for an acceptor over water on an equimolar basis. In this case, *S*_c_ was calculated to be 31, indicating a strong preference of the *Tr*Man5A-catalyzed reaction for 1-hexanol over water at the reaction conditions used. This is higher than *S*_c_ values for 1-hexanol in the range of 0.5–9 which have been observed with some other GHs (Adlercreutz 2017; Hansson and Adlercreutz 2001; Lundemo et al. 2017), but slightly lower than the *S*_c_ of 58 for 1-hexanol observed with the *Thermotoga neapolitana* β-glucosidase Bgl3B (Turner et al. 2007).

### Preparative synthesis and purification

In order to produce sufficient amounts of the identified hexyl mannooligosides for characterization, the reaction was scaled up. Here, a polymeric substrate, the linear INM polysaccharide, was used to obtain the donor saccharides. INM was pre-hydrolyzed into soluble mannooligosaccharides by *Pa*Man26A (Couturier et al. 2013), with M_4_ being the main oligosaccharide produced as analyzed with HPAEC-PAD (Table S1). Using this as donor substrate for alcoholysis with 1-hexanol, *Tr*Man5A produced 0.16 mM hexyl-M_2_ and 0.094 mM hexyl-M_3_ in an 8-h reaction. Here, the yield of the major alcoholysis product, hexyl-M_2_, was 0.6% based on initial M_4_ concentration. With preparative reversed-phase (C_18_) HPLC purification, a mixture consisting of 1.3 mg hexyl-M_2_ and 1.1 mg hexyl-M_3_ was obtained as a lyophilized powder.

### Structural characterization of hexyl mannooligosides

After having synthesized and purified a mixture of hexyl-M_2_ and hexyl-M_3_, their structures were characterized. First, they were analyzed with MALDI-TOF MS, and peaks corresponding to the masses of hexyl-M_2_ and hexyl-M_3_ were fragmented with MALDI-TOF/TOF tandem MS. The observed fragmentation masses were consistent with those expected from the predicted structures (Fig. 5). Hexyl-M_2_ fragmented into M_2_ (–H_2_O) and hexyl-M_1_, while hexyl-M_3_ also fragmented into M_3_ (–H_2_O) and hexyl-M_2_ in addition to the above two fragments (Fig. S3).

**Fig. 6:**
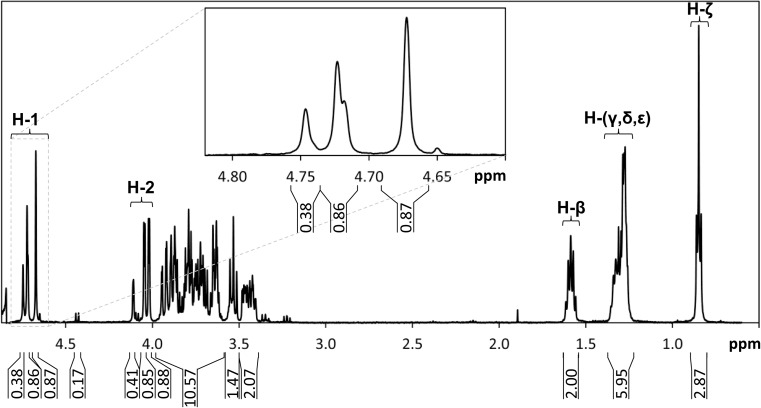
^1^H NMR spectrum of the synthesized hexyl mannooligosides. Peaks corresponding to anomeric protons (H-1) and C-2 protons (H-2) as well as protons on the β, γ, δ, ε, and ζ carbons of the hexyl chain (H-β, H-(γ,δ,ε), and H-ζ) are indicated within brackets. Integral values for different peaks relative to the H-β peak are shown beneath the *x*-axis. The insert shows an enlargement of the region of the spectrum containing the three H-1 shifts as indicated with the dashed lines

To obtain more detailed structural information, the hexyl mannooligoside mixture was analyzed with NMR. In ^1^H spectra collected at 10 °C, chemical shifts corresponding to anomeric protons (H-1) of three mannosyl units were observed at 4.67, 4.72, and 4.75 ppm and C-2 protons (H-2) at 4.0–4.2 ppm (Fig. 6). The chemical shifts at 4.72 and 4.75 ppm are in good agreement with H-1 shifts in terminal and internal β-mannosyl units reported for β-mannooligosaccharides previously (Harjunpää et al. 1995). Furthermore, with edited HSQC, the protons on the carbon of the hexyl –CH_2_ group adjacent to an oxygen atom can also be identified (Fig. S4). The carbon on this –CH_2_ group was observed to be coupled to the anomeric proton at 4.67 ppm in HMBC spectra, thus confirming bonding of the hexyl to that anomeric position (Fig. S4). No other chemical shift of anomeric protons showed such a correlation, and our conclusion is that both mannobiosides and mannotriosides in the sample have their anomeric proton chemical shift at 4.67 ppm.

Additionally, with ^1^H NMR at 25 °C, a coupling constant between H-1 and H-2 (*J*_1,2_) of 0.8 Hz was observed based on H-2 peak splitting on the hexyl-substituted mannosyl units (Fig. S5), which is consistent with previously reported *J*_1,2_ values of ~ 1 Hz for β-mannosyl H-1*s* (Harjunpää et al. 1995; Lundqvist et al. 2002). We also observed a coupling constant between H-2 and H-3 (*J*_2,3_) of 3.1 Hz (Fig. S5).

Since internal β-mannosyl units coupled to an adjacent mannosyl are only present in hexyl-M_3_, whereas terminal β-mannosyl units exist in both hexyl-M_2_ and hexyl-M_3_, the peak integral ratio of internal H-1 at 4.75 ppm to that of terminal H-1 at 4.72 ppm gives the fraction of hexyl mannooligosides that are hexyl-M_3_. In the ^1^H NMR spectrum (Fig. 6), the ratio is 38:86. From this ratio, the amounts of hexyl-M_2_ and hexyl-M_3_ were determined to be 0.43 and 0.47 mg, respectively, using hexyl CH_2_ quantification based on reference samples of hexyl-β-d-maltoside. The same sample was also quantified with HPLC using hexyl-β-d-maltoside as standard, with the amounts of hexyl-M_2_ and hexyl-M_3_ determined to be 0.57 and 0.48 mg, respectively.

### Determination of critical micelle concentration

**Fig. 7:**
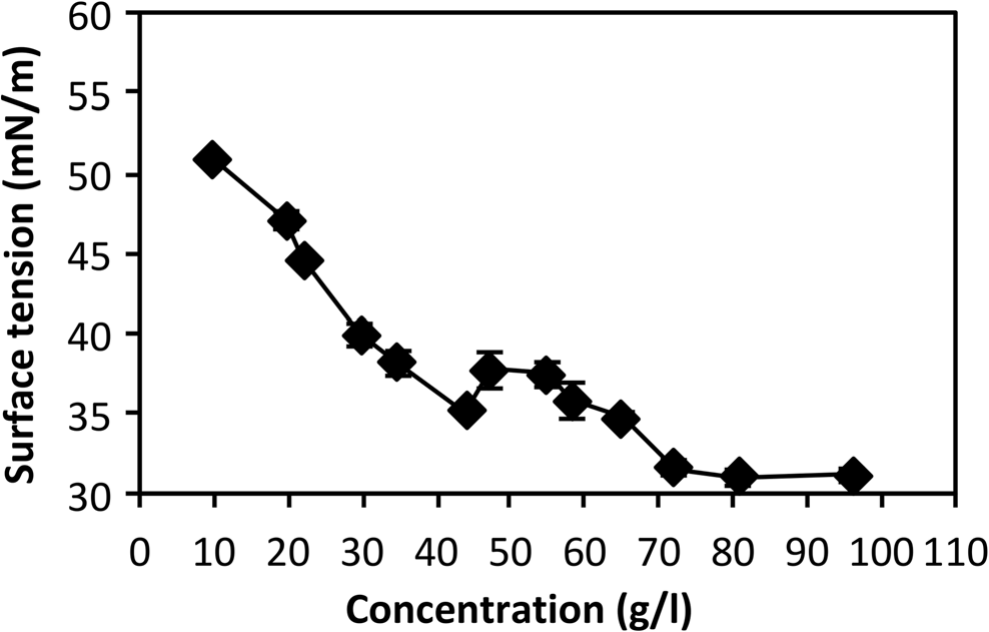
Surface tension plot of the purified hexyl mannooligoside mixture as determined with drop shape tensiometry, showing two breakpoints around 44 g/L (90 mM) and 72 g/L (147 mM). All data points are averages of at least two measurements. Error bars indicate standard deviations

To evaluate the surfactant properties of the purified hexyl mannooligosides, the critical micelle concentration (CMC) of the hexyl mannooligoside mixture was determined by means of surface tension measurements at different surfactant concentrations. The CMC was estimated from the breakpoint in the surface tension versus surfactant concentration curve (Dominguez et al. 1997). The hexyl mannooligoside surface tension curve indicates two breakpoints around 44 g/L (90 mM) and 72 g/L (147 mM) (Fig. 7), suggesting that the CMCs for the individual hexyl mannooligosides (hexyl-M_2_ and hexyl-M_3_) are in this region. For reference, surface tension measurements were also conducted with hexyl-β-d-glucoside and hexyl-β-d-maltoside individually and in mixtures with a molar ratio of 0.53:1. The results with two breakpoints at 26 g/L (82 mM) and 62 g/L (194 mM) in the surface tension curve of the reference mixture show the same trend as for the hexyl mannooligoside mixture (Fig. S6). The two breakpoints correspond to the CMCs of hexyl-β-d-glucoside (29 g/L or 110 mM) and hexyl-β-d-maltoside (59 g/L or 137 mM) determined separately (Fig. S6).

## Discussion

The study and application of β-mannanases is of fundamental importance for the utilization and valorization of plant biomass due to the high amounts of β-mannan in prevalent softwoods such as spruce (Lundqvist et al. 2002; Moreira and Filho 2008; Yamabhai et al. 2016). The present study describes the first reported instance of β-mannanase-catalyzed synthesis of alkyl mannosides with surfactant properties and opens up the possibility of using renewable β-mannans for enzymatic synthesis of surfactants. 1-Hexanol was used as acceptor, and polymeric β-mannan was used to prepare donor saccharides (mainly M_4_). Previous studies have demonstrated the alcoholytic capabilities of β-mannanases with shorter-chain primary alcohols such as methanol and 1-butanol (Rosengren et al. 2012; Rosengren et al. 2014). Transfer with 1-hexanol as acceptor has been demonstrated with other GHs such as β-mannosidases (Itoh and Kamiyama 1995), xylanases (Jiang et al. 2004), and β-glucosidases (Lundemo et al. 2013; Lundemo et al. 2014; Lundemo et al. 2017; Turner et al. 2007).

In the current study, relative MALDI-TOF MS peak areas of alcoholysis products and oligosaccharide products were used to estimate DA, reflecting the fraction of alcoholysis products formed in a reaction as explained in the “Materials and methods” section. MALDI-TOF MS is a good screening method for relative comparisons of enzymes and reaction conditions, although additional methods are needed for absolute quantification and the response cannot be expected to be the same for different types of molecules due to potential ion suppression in MALDI-TOF MS (Duncan et al. 2008). Often thin-layer chromatography is used to make an initial assessment of transglycosylation products (see, e.g., Jain et al. 2014). In the present paper, we show that MALDI-TOF MS analysis is an attractive alternative or a complementary screening method, which is at least as fast and gives additional information on the mass of products and thus more certain primary product identification.

In this study, the initial DA with 1-hexanol was significantly lower than with methanol for all three enzymes included in the present study, indicating that 1-hexanol is more difficult to use as acceptor in alcoholysis compared to methanol. Several other studies have demonstrated decreasing yields of alkyl glycosides with increasing alkyl chain lengths, a contributing factor being the low water solubility of longer-chain alcohols (Ochs et al. 2011; Turner et al. 2007). The accessibility and properties of the positive subsite region are another factor that may influence transglycosylation reactions using acceptors with longer alkyl chains (Durand et al. 2016; Ochs et al. 2013).

Enzyme comparison revealed differences in alcoholysis capacity between the studied enzymes. With 1-hexanol, *Tr*Man5A had the highest DA of the three β-mannanases (Table 1). Acceptor affinity in positive subsites is known to affect transglycosylation capacity (Rosengren et al. 2012; Rosengren et al. 2014; Tran et al. 2014). The R171K substitution in the + 2 subsite of *Tr*Man5A has previously been shown to eliminate transglycosylation with saccharides as acceptors but not alcoholysis with methanol (Rosengren et al. 2012). Therefore, it might be expected that the competing reaction (with saccharides as acceptors) could be diminished. It is interesting to note that a lower DA using 1-hexanol as acceptor was observed with *Tr*Man5A-R171K compared to *Tr*Man5A (Table 1). However, further investigation would be needed to elucidate if R171 would have any role in the usage of 1-hexanol as acceptor. The + 1 subsite of *An*Man5C contains a tryptophan, W283, that appears to facilitate transglycosylation with saccharides as acceptors (Dilokpimol et al. 2011), but here, a positive contribution to the usage of 1-hexanol as acceptor is unlikely since the DA was clearly lowest among the tested enzymes. Based on the comparably high DA for the wild-type *Tr*Man5A (Table 1) and its stability during prolonged incubations (Fig. 3), this enzyme was selected for further studies of hexyl mannooligoside synthesis.

Varying reaction conditions can also affect alcoholysis product yields. In alcoholysis with 1-hexanol and *Tr*Man5A, a lower enzyme concentration resulted in a moderate increase in DA (Fig. 3). Enzyme concentration has previously been shown to affect the observed ratio of hydrolysis products versus transglycosylation products, with a lower enzyme load favoring transglycosylation products (Guo et al. 2016; Manas et al. 2014), possibly due to increased secondary product hydrolysis at higher enzyme loads. The slight decrease in DA over time observed with the highest *Tr*Man5A concentration used could possibly be the result of secondary hydrolysis of hexyl mannooligosides (Fig. 3). The concentration of the donor substrate (M_4_ in this case) also affected alcoholysis, where a similar DA but a higher rate of M_4_ conversion was observed with 25 mM M_4_ compared to 5 mM (Table 2). Twenty-five millimolars of M_4_ was therefore used in subsequent reactions. However, increasing the M_4_ concentration too much appears to reduce alcoholysis, since a lower DA was observed with 50 mM M_4_. Since oligosaccharides with DP 5–9 were detected in the 50-mM reaction, this can possibly be due to transglycosylation with saccharides as acceptors competing with alcoholysis at higher M_4_ concentrations, similar to the observed effect of transglycosylation on hydrolysis in, e.g., β-glucosidases (Bohlin et al. 2013). Higher substrate concentrations, in general, are expected to increase transglycosylation (Sinnott 1990) as exemplified with, e.g., a retaining GH5 β-mannosidase (Dias et al. 2004). The R171K substitution in the + 2 subsite of *Tr*Man5A (Rosengren et al. 2012) may still be valuable at higher donor saccharide concentrations where oligosaccharide elongation would be more effective (Biely et al. 1981; Sinnott 1990), in line with products of DP 5–9 being detected with 50 mM M_4_ for *Tr*Man5A.

In the scaled-up reaction, a polymeric β-mannan (INM) was pre-hydrolyzed into mainly M_4_ by *Pa*Man26A (Couturier et al. 2013) and then used as donor substrate. Using polymeric substrates for enzymatic synthesis represents a step towards β-mannan utilization in biorefineries (Cherubini 2010). The lower yield of alcoholysis products observed with the pre-hydrolyzed INM compared to reactions with M_4_ could be partially due to the presence of lower amounts of other oligosaccharides (M_2_ and M_3_) in the hydrolysate (Table S1), which might act as acceptors for transglycosylation and thereby possibly compete with alcoholysis as described above (Bohlin et al. 2013).

We successfully managed to purify (Fig. 4) and characterize the synthesized hexyl mannooligosides. The expected structures, hexyl β-mannobioside and hexyl β-mannotrioside (Fig. 5), are supported by the MALDI-TOF MS/MS (Fig. S3) and NMR data (Fig. 6, Fig. S4-S5). The surfactant properties of the purified hexyl mannooligosides were also determined by tensiometry. Two breakpoints in the surface tension versus concentration curve were observed (Fig. 7), which suggests that the solution is a mixture of two different types of surfactants with different surface activities, in line with the concluded chemical composition. This phenomenon has previously been observed for mixtures of alkyl polyglucosides with different alkyl chain lengths (Balzer and Luders 2000). Reference experiments with hexyl-β-d-glucoside and hexyl-β-d-maltoside, with the same alkyl chain length but different polar head groups, support this observation (Fig. S6). The existence of a minimum around the first breakpoint is consistent with the solubilization of the more hydrophobic (or surface-active) surfactant in the micelles when the total surfactant concentration increases (Lin et al. 1999).

In conclusion, β-mannanase-catalyzed synthesis of hexyl mannooligosides with surfactant properties has been demonstrated for the first time, using the *Trichoderma reesei* GH5 β-mannanase *Tr*Man5A. Hexyl mannooligosides were synthesized from β-mannan and 1-hexanol and purified using preparative HPLC. Their surfactant properties were evaluated, showing similar CMC values compared to commercially available alternatives. Future studies could involve protein engineering which is a strategy with potential to increase transglycosylation rate and/or yield (Lundemo et al. 2013; Lundemo et al. 2017). In some cases, subsite − 1 residues have been substituted, shown to be an applicable route when using activated (nitrophenyl) donor sugars and *exo*-glycosidases (Bissaro et al. 2014; Teze et al. 2015; Teze et al. 2014). When using natural non-activated donor sugars, as in the present study, another approach would potentially be needed. For β-mannanases (Dilokpimol et al. 2011; Rosengren et al. 2012) and other GHs (Armand et al. 2001; Feng et al. 2005), positive subsites have been shown to be important for saccharide acceptor interactions and thus influence transglycosylation capacity. Assuming that acceptor interaction is important also for longer-chain alcohols, the properties of these alcohols would imply that introduction of hydrophobic residues within positive subsites could be beneficial for efficient transglycosylation (Durand et al. 2016). Hydrophobic residues may also, in certain cases, reduce water accessibility and lower hydrolysis (Kuriki et al. 1996). Future work with β-mannanases and long-chain acceptors could involve identification of further positive subsite residues as targets for protein engineering.

## Electronic supplementary material

ESM 1 (PDF 640 kb)
