# Supplementary material for: β-Mannanase-catalyzed synthesis of alkyl mannooligosides
Source: Appl Microbiol Biotechnol. 2018 Apr 22;102(12):5149–63. doi: 10.1007/s00253-018-8997-2 (PMC5959982; doi:10.1007/s00253-018-8997-2)
Supplement: Supplementary file 1 — (PDF 640 kb) [file 253_2018_8997_MOESM1_ESM.pdf]

Electronic supplementary material
Applied Microbiology and Biotechnology

β-Mannanase-catalyzed synthesis of alkyl mannooligosides

Johan Morrill[a], Anna Månberger, Anna Rosengren[a], Polina Naidjonoka[c], Pernille von Freiesleben[d], Kristian B.R.M. Krogh[d], Karl-Erik Bergquist[e], Tommy Nylander[c], Eva Nordberg Karlsson[b], Patrick Adlercreutz[b], Henrik Stålbrand[a*]

[a] Biochemistry and Structural Biology, [b] Biotechnology, [c] Physical Chemistry, [e] Centre for Analysis and Synthesis, Department of Chemistry, Lund University, PO Box 124, S-221 00, Lund, Sweden. [d] Novozymes A/S, Krogshøjvej 36, 2880 Bagsværd, Denmark.
[*] Corresponding author. Phone: +46-46-2228202. E-mail: henrik.stalbrand@biochemistry.lu.se

Table S1. Concentrations of mannooligosaccharides in the hydrolyzed INM used as donor substrate in the scaled-up alcoholysis reaction, as determined with HPAEC-PAD.

**Oligosaccharide**	**M_1**	**M_2**	**M_3**	**M_4**
Concentration (mM)	1.87 ± 0.29	0.86 ± 0.13	0.80 ± 0.13	2.10 ± 0.43

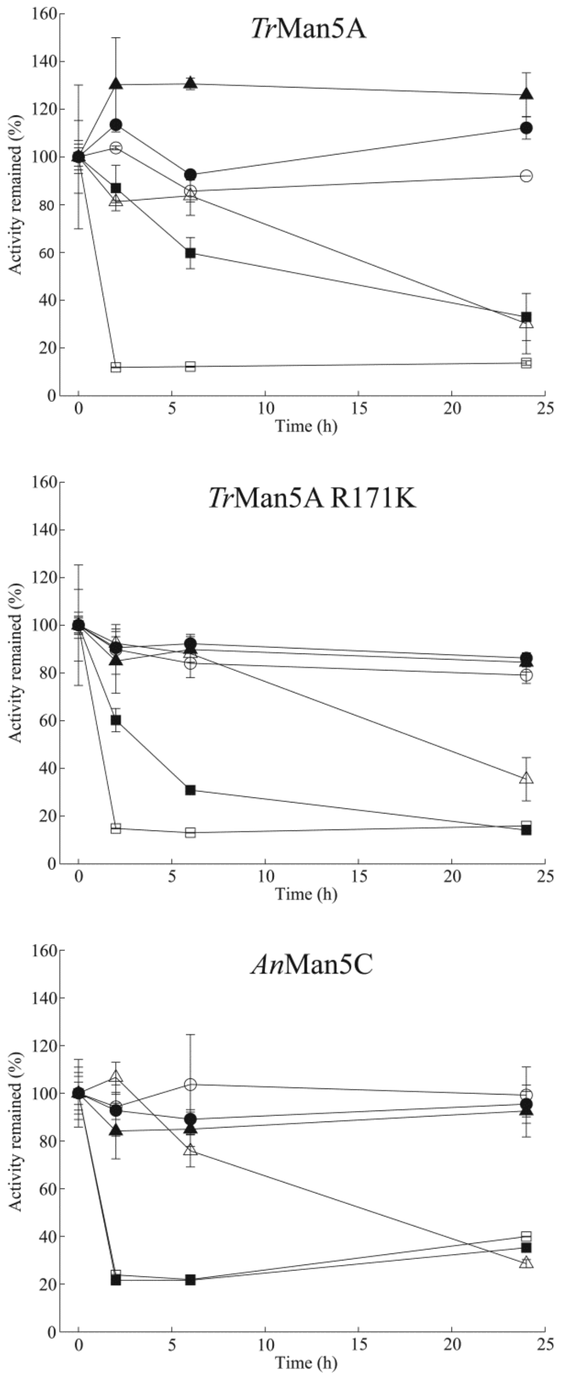

Figure S1. Stability of enzymes in several concentrations of methanol and 1-hexanol at 37°C. ● - No alcohol, ○ - 25% methanol, ▪ - 50% methanol, ▫ - 75% methanol, ▲ - 5% 1-hexanol, △ - 25% 1-hexanol. Activity assay performed at 37°C for 10 minutes. Each point is the average of at least two samples and the error bars represent standard deviations.

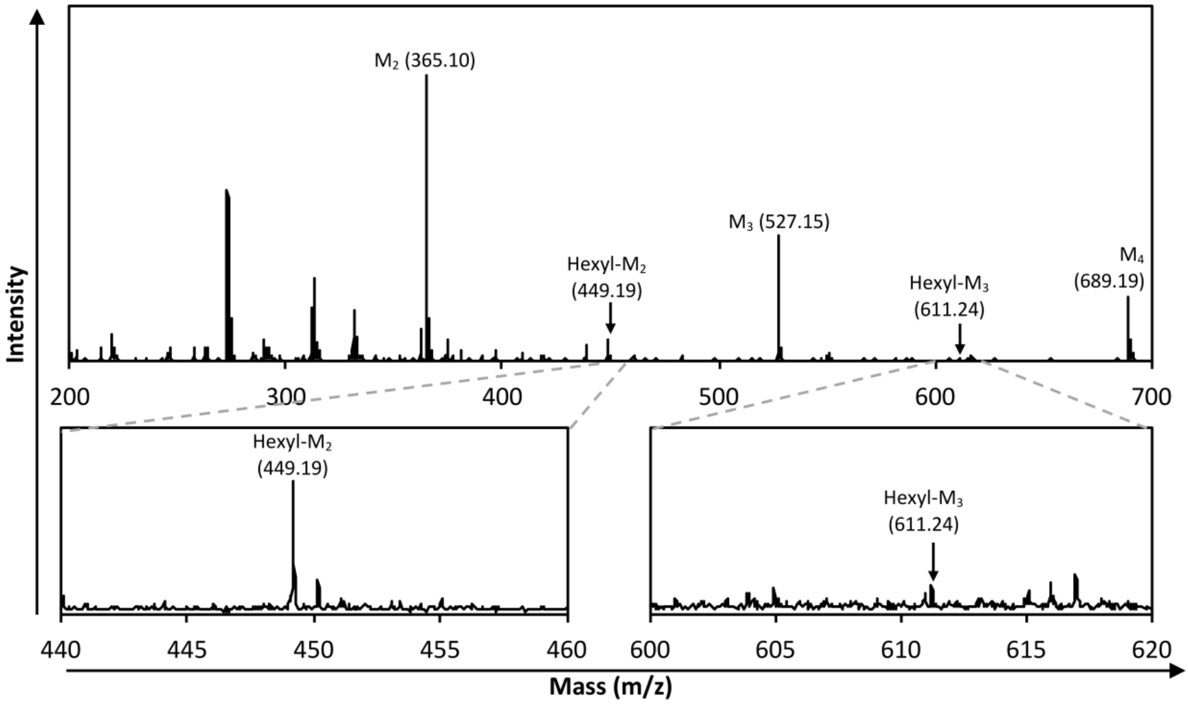

Figure S2. MALDI-TOF MS spectra of alcoholysis reaction after 1 hour with 2 µM *Tr*Man5A, 5 mM M_4 and 25% (v/v) 1-hexanol. Peaks correspond to experimentally determined monoisotopic masses of sodium adducts of present mannooligosaccharides and hexyl mannooligosides.The theoretical monoisotopic sodium adduct masses of these compounds are: M_2, 365.11; M_3, 527.16; M_4, 689.21; hexyl-M_2, 449.20; and hexyl-M_3, 611.25.

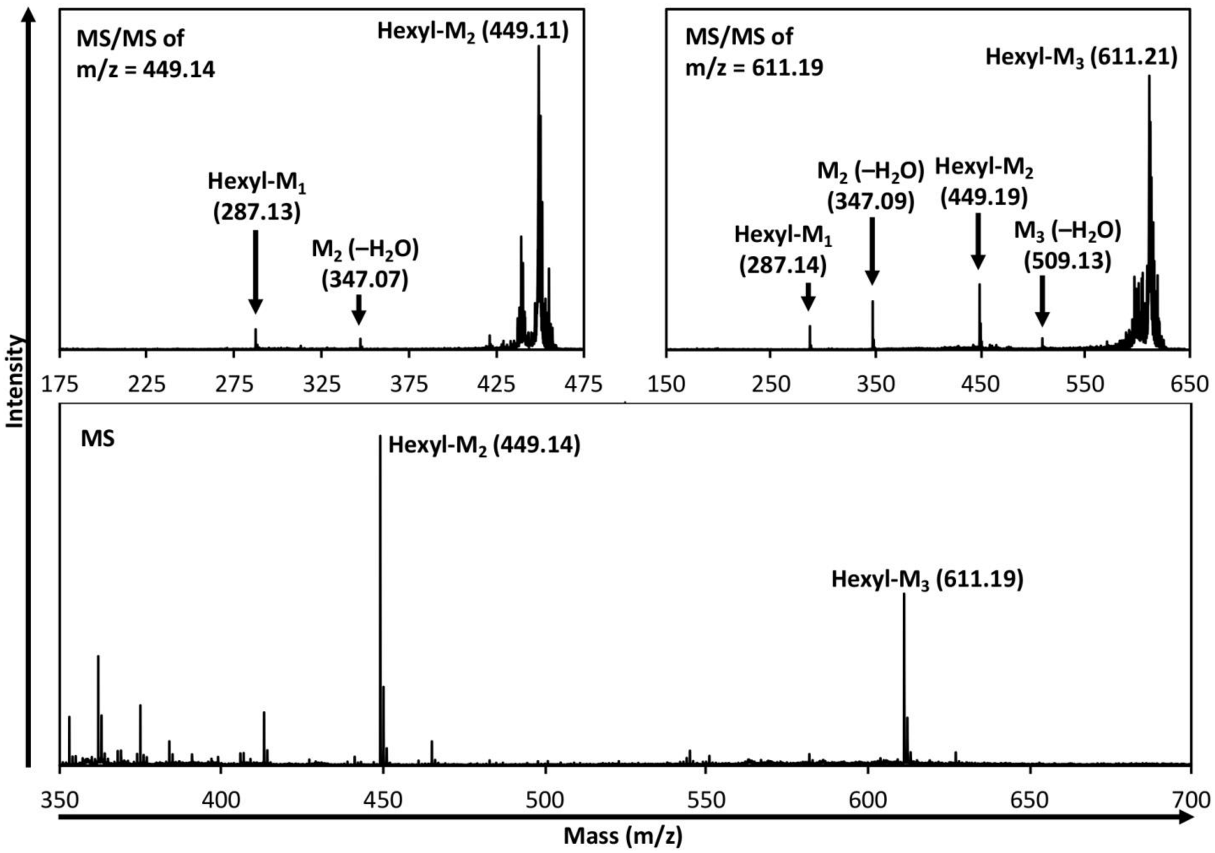

Figure S3. MALDI-TOF MS and MS/MS of hexyl-M_2 and hexyl-M_3, showing fragmentation patterns consistent with the predicted structures of each hexyl mannooligoside. Numbers in parentheses indicate the experimental monoisotopic sodium adduct masses of each peak. The theoretical monoisotopic sodium adduct masses of these compounds are: hexyl-M_1, 287.15; hexyl-M_2, 449.20; hexyl-M_3; 611.25; M_2 (–H_2O), 347.10; M_3 (–H_2O), 509.15.

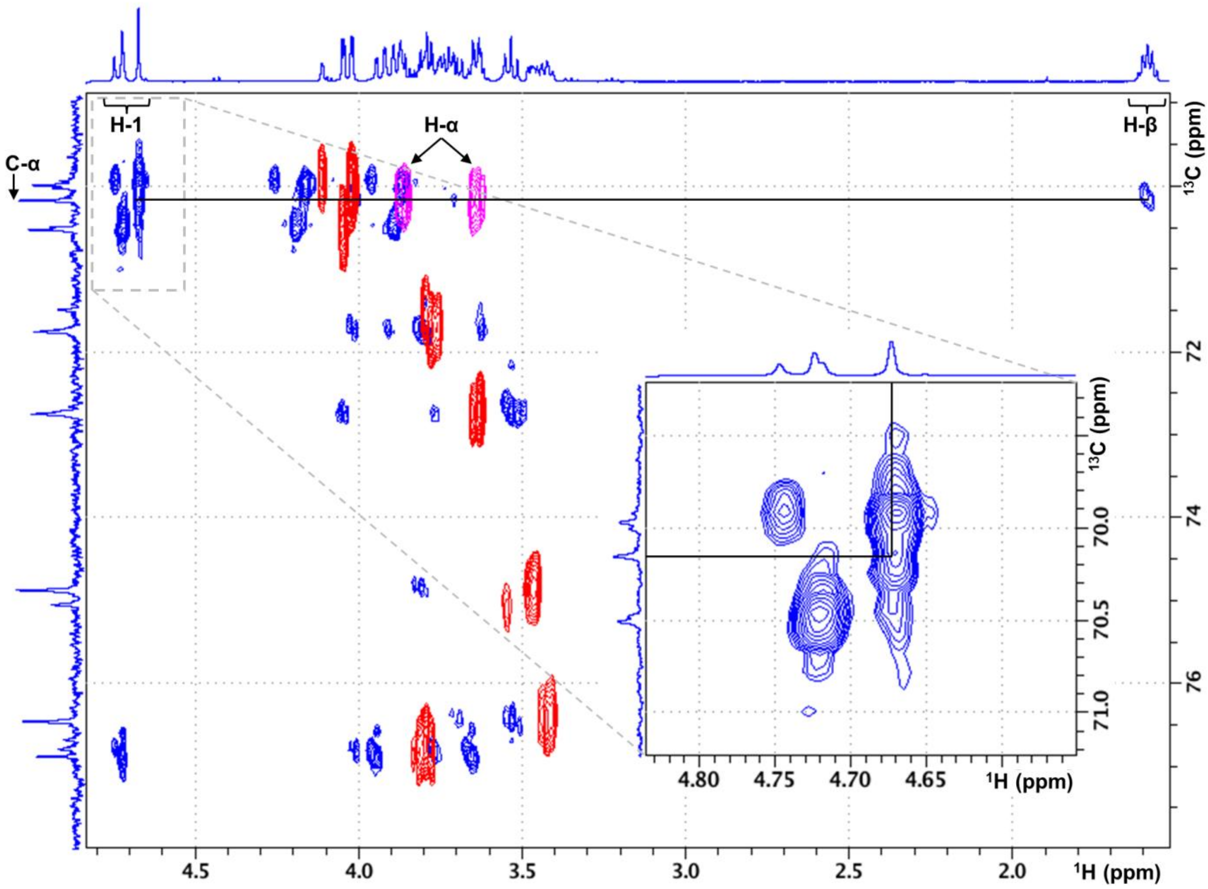

Figure S4. Overlay of ^{1}H-^{13}C HMBC (blue, positive signals) and edited HSQC (red, positive signals; magenta, negative signals) NMR spectra of the synthesized hexyl mannooligosides. The horizontal line is drawn at the chemical shift of the hexyl C-α carbon at 70.1 ppm, Along that line a long-range J-coupling to the hexyl H-β protons is shown with a blue crosspeak at 1.6 ppm, direct bond J-coupling to H-α protons from crosspeaks in magenta at 3.6 and 3.8 ppm and (as shown in the insert) a long-range J-coupling from the hexyl C-α carbon at 70.1 ppm to the anomeric proton at 4.67 ppm.

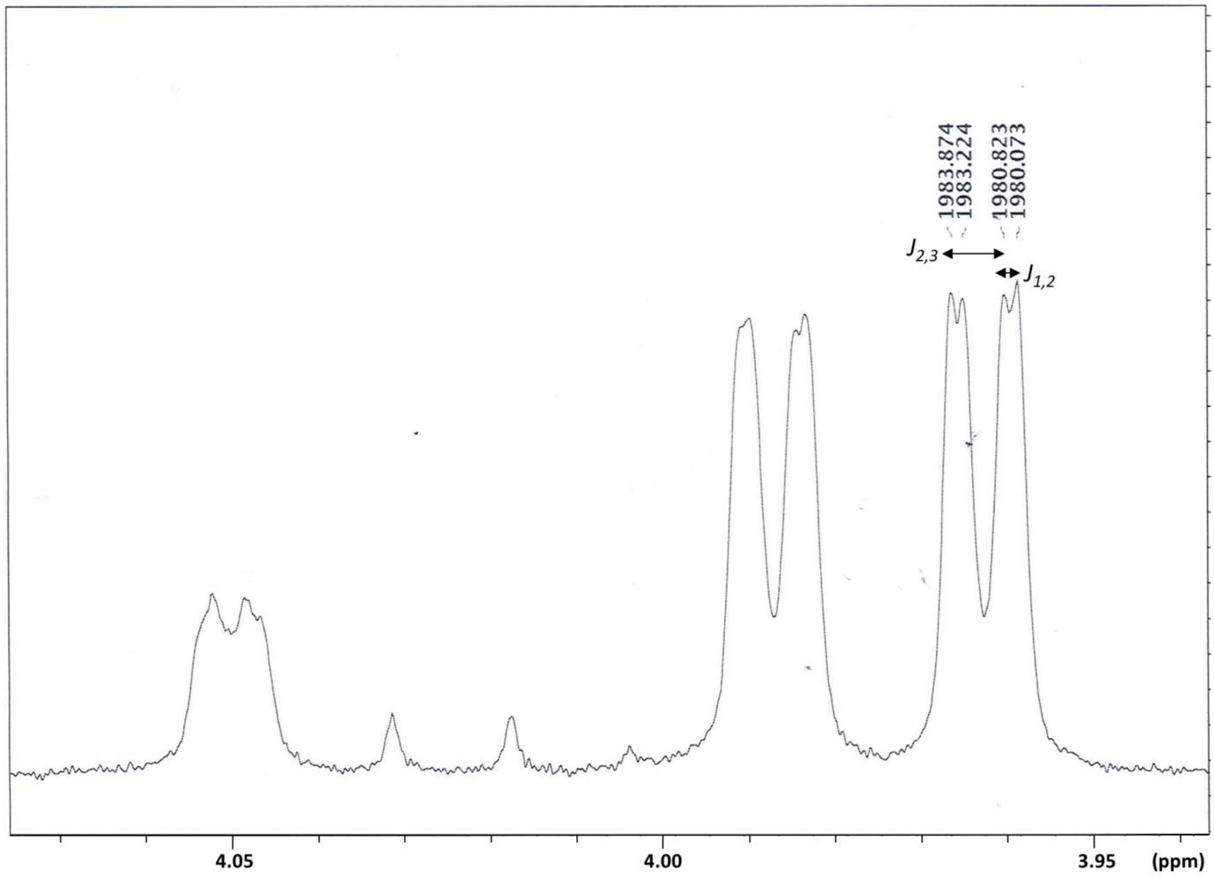

Figure S5. ^{1}H NMR spectrum of the synthesized hexyl mannooligosides collected at 25°C, showing H-2 peak splitting. Peak picking for four peaks indicating the H1-H2 coupling constant ($J_{1,2}$) and the H2-H3 coupling constant ($J_{2,3}$) are shown in Hz above the peaks. A $J_{1,2}$ of 0.8 Hz (1980.823 – 1980.073) and a $J_{2,3}$ of 3.1 Hz (1983.874 – 1980.823) can be observed as indicated by the arrows. The obtained $J_{1,2}$ value is consistent with such previously reported for β-mannosyls (see article running text), although to conclude β-configuration from NMR data would have required determination of the C1-H1 coupling constant.

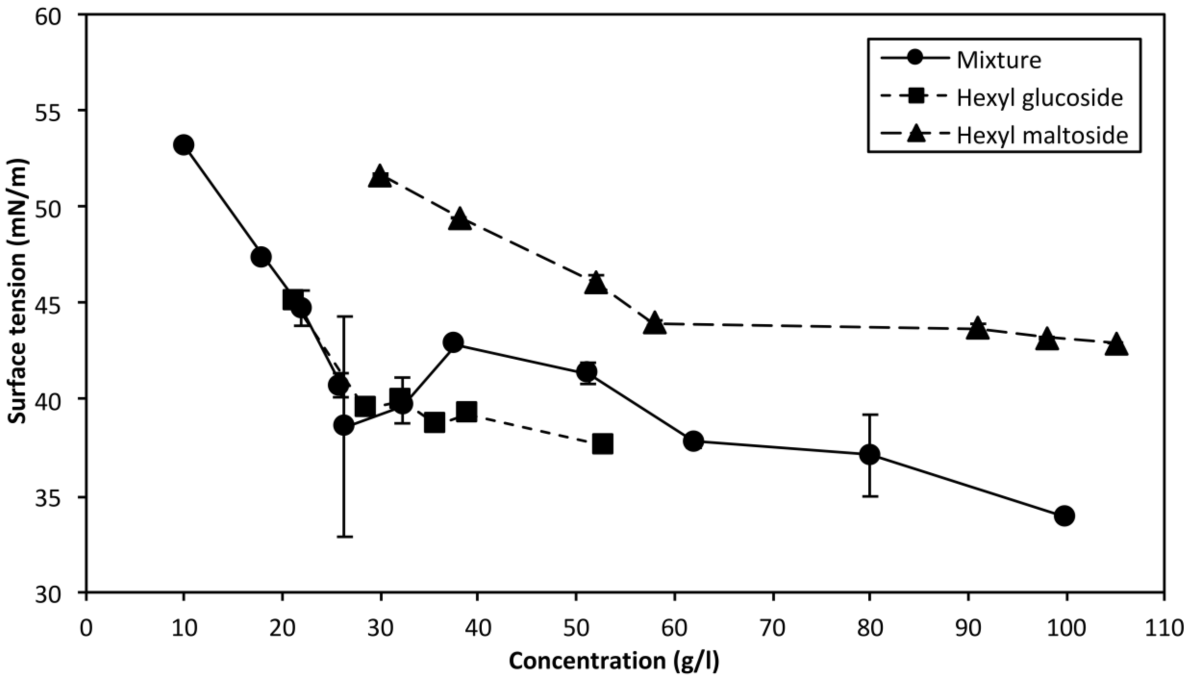

Figure S6. Surface tension plots of hexyl β-D-glucoside (squares, short dashes) and hexyl β-D-maltoside (triangles, long dashes) separately, as well as a mixture of a molar ratio of 0.53:1 of hexyl β-D-glucoside and hexyl β-D-maltoside (circles, solid line), determined with drop shape tensiometry. CMCs of 29 g/L (110 mM) for hexyl β-D-glucoside and 59 g/L (137 mM) for hexyl β-D-maltoside are indicated by the breakpoint in the respective data series. In the mixture of hexyl β-D-glucoside and hexyl β-D-maltoside, two breakpoints can be observed around 26 g/L (82 mM) and 62 g/L (194 mM). All data points are averages of at least two measurements. Error bars indicate standard deviations.
